# Investigation of Acoustic Cardiographic Parameters before and after Hemodialysis

**DOI:** 10.1155/2019/5270159

**Published:** 2019-11-05

**Authors:** Hui-Ju Tsai, Yi-Chun Tsai, Jiun-Chi Huang, Pei-Yu Wu, Szu-Chia Chen, Yi-Wen Chiu, Jer-Ming Chang, Hung-Chun Chen

**Affiliations:** ^1^Department of Family Medicine, Kaohsiung Municipal Ta-Tung Hospital, Kaohsiung Medical University Hospital, Kaohsiung Medical University, Kaohsiung, Taiwan; ^2^Graduate Institute of Clinical Medicine, College of Medicine, Kaohsiung Medical University, Kaohsiung, Taiwan; ^3^Division of Nephrology, Department of Internal Medicine, Kaohsiung Medical University Hospital, Kaohsiung Medical University, Kaohsiung, Taiwan; ^4^Faculty of Medicine, College of Medicine, Kaohsiung Medical University, Kaohsiung, Taiwan; ^5^Department of Internal Medicine, Kaohsiung Municipal Hsiao-Kang Hospital, Kaohsiung Medical University, Kaohsiung, Taiwan

## Abstract

Patients with end-stage renal disease are at an increased risk of cardiovascular diseases and associated mortality. Acoustic cardiography is a technique in which cardiac acoustic data is synchronized with electric information to detect and characterize heart sounds and detect heart failure early. The aim of this study was to investigate acoustic cardiographic parameters before and after hemodialysis (HD) and their correlations with ankle-brachial index (ABI), brachial-ankle pulse wave velocity (baPWV), and ratio of brachial preejection period to ejection time (bPEP/bET) obtained from an ABI-form device in HD patients. This study enrolled 162 HD patients between October 2016 and April 2018. Demographic, medical, and laboratory data were collected. Acoustic cardiography was performed before and after HD to assess parameters including third heart sound (S3), fourth heart sound (S4), systolic dysfunction index (SDI), electromechanical activation time (EMAT), and left ventricular systolic time (LVST). The mean age of the enrolled patients was 60.4 ± 10.9 years, and 86 (53.1%) patients were male. S4 (*p* < 0.001) and LVST (*p* < 0.001) significantly decreased after HD, but EMAT (*p* < 0.001) increased. Multivariate forward linear regression analysis showed that EMAT/LVST before HD was negatively associated with albumin (unstandardized coefficient *β* = ‐0.076; *p* = 0.004) and ABI (unstandardized coefficient *β* = ‐0.115; *p* = 0.011) and positively associated with bPEP/bET (unstandardized coefficient *β* = 0.278; *p* = 0.003). Screening HD patients with acoustic cardiography may help to identify patients at a high risk of malnutrition, peripheral artery disease, and left ventricular systolic dysfunction.

## 1. Introduction

End-stage renal disease (ESRD) is a serious health care issue, and patients with ESRD are at an increased risk of cardiovascular disease and mortality [1, 2]. In addition, cardiac structural and functional abnormalities and traditional risk factors such as hypertension, age, diabetes, and dyslipidemia are also known to increase the risk of cardiovascular disease [3, 4]. Accordingly, the early identification of these risk factors in these patients is crucial.

Left ventricular (LV) dysfunction is highly prevalent in ESRD patients, and LV dysfunction is an important factor predisposing ESRD patients to sudden death [5–7]. LV ejection fraction and geometry-independent methods such as midwall fractional shortening (mwFS) can be used to estimate LV systolic function [8]. Echocardiography is the most commonly used tool to assess cardiac function; however, its efficacy depends on the operator's ability and it is time-consuming [9]. Audicor acoustic cardiography is a simple technique in which cardiac acoustic data is synchronized with electrocardiography recordings to provide a comprehensive assessment of both mechanical and electronic functions of the heart [10]. Previous studies have reported that acoustic cardiography can be used as a noninvasive method to detect LV systolic dysfunction [11–17] and increase LV filling pressure [18, 19]. Acoustic cardiography has also been used to detect heart failure and ischemic heart disease, LV hypertrophy, constrictive pericarditis, sleep apnea, and ventricular fibrillation [10].

The third heart sound (S3) is caused by an abrupt limitation of LV inflow during early diastole that causes vibration of the cardiohemic system [20]. The abnormal S3 has been considered to be a specific clinical sign for LV dysfunction and a predictor for poor prognosis of patients with heart failure [21]. The fourth heart sound (S4) is produced by the abrupt deceleration of the A wave [20]. The presence of S4 was correlated with LV stiffness, impaired relaxation, and stress-induced ischemia [20, 22, 23]. Systolic time intervals (STIs) have been validated to predict LV dysfunction in previous studies [24, 25].

Arterial stiffness, peripheral artery disease (PAD), and LV systolic dysfunction are known to contribute to LV hypertrophy and cardiovascular risks [26–30]. The ABI-form (Colin VP1000, Komaki, Japan) clinical device simultaneously measures the blood pressure in both ankles and arms and records brachial and posterior tibial artery pulse waves using an automated oscillometric method. This device can thus be used to acquire brachial-ankle pulse wave velocity (baPWV) and ankle-brachial index (ABI), both of which have been shown to be good markers for arterial stiffness and PAD, respectively [31, 32]. In addition, the ABI-form device can be used to calculate the brachial preejection period (bPEP) and brachial ejection time (bET) according to electrocardiographic, phonocardiographic, and brachial pressure volume waveform signals [31]. We recently found a significant correlation between bPEP/bET and LVEF and that is a useful marker to predict impaired LV systolic function [33, 34].

The timely identification and treatment of hemodialysis (HD) patients with LV dysfunction may improve the prognosis of ESRD. To the best of our knowledge, acoustic cardiography has not been previously investigated in HD patients. Moreover, no previous study has investigated changes in acoustic cardiographic markers before and after HD. Therefore, the aim of this study was to investigate acoustic cardiographic parameters in HD patients before and after HD and correlations between ABI, baPWV, and bPEP/bET obtained from an ABI-form device in HD patients.

## 2. Materials and Methods

### 2.1. Study Patients and Design

We performed this study at the Dialysis Unit of Kaohsiung Medical University Hospital (KMUH), a regional hospital in the south part of Taiwan, from October 2016 to April 2018, and included patients who received HD treatment three times per week, with each session lasting 3.5 to 4 hours. The blood flow rate of the dialyzer ranged from 250 to 300 mL/min with a dialysate flow rate of 500 mL/min. In total, we enrolled 162 maintenance HD patients into this study. This study was approved by the Institutional Review Board of KMUH, and all of the patients provided informed consent.

### 2.2. Demographic, Medical, and Laboratory Data Collection

We obtained data on sex, age, current smoking status, and comorbidities from interviews and medical records. Blood samples were collected following overnight fasting and within 1 month of study enrollment to measure various biomarkers including albumin, fasting glucose, triglycerides, total cholesterol, hemoglobin, creatinine, total calcium, phosphorus, and uric acid. The ultrafiltration rate was defined as the ultrafiltration/body weight, and the efficiency of dialysis was determined by Kt/V according to the Daugirdas method [35].

### 2.3. Assessment of ABI, baPWV, bPEP, and bET

The detailed methods have been described in previous studies [36–38]. In brief, we used an ABI-form device to determine ABI and baPWV 30 minutes before each HD session. The device simultaneously measured the blood pressure in the patients' arms and ankles using an oscillometric method [31, 38, 39]. The patients were placed in the supine position, and occlusion and monitoring cuffs were placed tightly around the upper arms and both sides of the lower extremities without vascular access after 10 minutes of rest. ABI was determined as the systolic blood pressure in the ankle divided by that in the arm, and the lowest systolic blood pressure in the ankle was used in the calculations. Brachial and tibial artery pulse waves were used to calculate baPWV and the transmission time, which was defined as the time period between the initial increases in brachial waveform and tibial waveform. Transmission distance was calculated as the distance between the ankle and arm using the body height of the patient. baPWV was determined using the ABI-form device as the transmission distance divided by the transmission time, with the highest baPWV value being used. The ABI-form device also determined bPEP and bET values, with bET being determined from the foot to dicrotic notch of the brachial pulse volume recording (equivalent to the incisura on the downstroke of the aortic pressure wave contour produced by closure of the aortic valve) [38]. The whole electromechanical systolic interval was calculated from the onset of the Q wave as recorded in electrocardiography to the first high-frequency vibration of the aortic component of the second heart sound (S2) in phonocardiography. bPEP was calculated as electromechanical systolic interval − bET [9, 33, 34].

### 2.4. Acoustic Cardiography

Each patient received the acoustic cardiographic examination in the supine position 30 minutes before HD and 30 minutes after HD. Acoustic cardiographic data and ABI were measured in the same month. Acoustic cardiographic sensors were placed two sensors on the V3 and V4 positions of the chest. Two standard electrodes were attached on the limb which leads the position of standard electrocardiography. Acoustic cardiographic data were transferred to an AUDICOR® system (Inovise Medical), on which heart sounds and related systolic time intervals were analyzed. The following acoustic cardiographic parameters were measured in this study [10, 40]:

1. Electromechanical activation time (EMAT) and %EMAT—the time from the Q wave onset to the mitral component of the first heart sound (S1): EMAT represented the time required for the left ventricle to generate sufficient force to close the mitral valve. Percent EMAT (%EMAT) was computed as the EMAT divided by the dominant R-R interval
2. LV systolic time (LVST) and %LVST—the time from S1 to S2: percent LVST (%LVST) was computed as the LVST divided by the dominant R-R interval
3. The third heart sound (S3)—the probability that S3 exists: one value between 0 and 10 was reported, and a value ≥ 5 was used to define the presence of S3
4. The fourth heart sound (S4)—similar to S3: one value from 0 to 10 was reported, with a value ≥ 5 defining the presence of S4
5. Systolic dysfunction index (SDI): SDI was derived from the nonlinear transformation of [(S3 score ÷ 10) × QRS duration × QR interval × %EMAT] and mapped onto a scale of 0 to 10

### 2.5. Statistical Analysis

Data are expressed as percentages and mean ± standard deviation for acoustic cardiographic parameters and median (25th–75th percentile) for triglyceride level and dialysis duration. All of the patients were classified into two groups by a median EMAT/LVST value. Differences between groups were assessed using the chi-square test for categorical variables, the independent *t*-test for continuous variables with approximately normal distribution, and the Mann-Whitney *U* test for continuous variables with skewed distribution. The paired *t*-test was used to compare acoustic cardiographic parameters before and after HD. Multiple forward linear regression analysis was used to identify the factors associated with EMAT/LVST, EMAT, and LVST before HD. Forward selection involves starting with no variables in the model, testing the addition of each variable using a chosen model fit criterion, adding the variable (if any) whose inclusion gives the most statistically significant improvement of the fit, and repeating this process until none improves the model to a statistically significant extent. A difference was considered significant if the *p* value was less than 0.05. Statistical analyses were performed using SPSS 18.0 for Windows (SPSS Inc. Chicago, USA).

## 3. Results

The mean age of the 162 HD patients included in this study was 60.4 ± 10.9 years, and 86 (53.1%) were male. The median EMAT/LVST value was 0.27, and the patients were stratified into two groups according to whether their EMAT/LVST value was ≤0.27 (group 1; *n* = 81) or >0.27 (group 2; *n* = 81). A comparison of the clinical characteristics between the study groups is shown in Table 1. Compared to the patients in group 1, those in group 2 had a higher hemoglobin level and high SDI before HD.

Changes in acoustic cardiographic parameters before and after HD are shown in Table 2. S4 (*p* < 0.001) and LVST (ms and %) (*p* < 0.001) significantly decreased after HD; however, EMAT (*p* < 0.001) significantly increased after HD in all patients. In group 2, S4, the prevalence of S4 ≥ 5, and LVST (%) (*p* < 0.001) significantly decreased after HD; however, there was no difference in SDI after HD. In group 1, S4 and LVST (ms and %) significantly decreased after HD (*p* < 0.001); however, EMAT (ms and %) significantly increased after HD (*p* < 0.001). S3 and the proportion of S3 ≥ 5 had the statistical tendency to decrease after HD, but they did not achieve significance.

Table 3 displays the unstandardized coefficients (*β*) of EMAT/LVST before HD after adjusting for age, sex, smoking habit, a history of diabetes, hypertension, coronary artery disease, cerebrovascular disease, systolic blood pressure, diastolic blood pressure, body mass index, ABI, baPWV, bPEP/bET, duration of HD, albumin, fasting glucose, triglycerides, total cholesterol, hemoglobin, creatinine, calcium-phosphorous product, uric acid, Kt/V, and ultrafiltration percent. In forward multivariate analysis, EMAT/LVST before HD was negatively associated with serum albumin level (unstandardized *β* = ‐0.076, *p* = 0.004) and ABI (unstandardized *β* = ‐0.115, *p* = 0.011) and positively associated with bPEP/ET (unstandardized *β* = 0.278, *p* = 0.003). EMAT was negatively associated with serum albumin level (unstandardized *β* = ‐17.976, *p* = 0.001) and positively associated with bPEP/bET (unstandardized *β* = 53.879, *p* = 0.004). LVST was positively associated with ABI (unstandardized *β* = 92.032, *p* < 0.001) and negatively associated with bPEP/bET (unstandardized *β* = ‐122.771, *p* = 0.005). Because the baPWV was frequently underestimated and also deteriorated by PAD in the hemodialysis population [41], we analyzed the data excluding ABI ≤ 0.9 and these results are shown in Table 4. After excluding ABI ≤ 0.9, EMAT/LVST before HD was negatively associated with serum albumin level (unstandardized *β* = ‐0.042, *p* = 0.095) and creatinine (unstandardized *β* = ‐0.006, *p* = 0.048) and positively associated with bPEP/ET (unstandardized *β* = 0.308, *p* < 0.001) and BMI (unstandardized *β* = 0.006, *p* < 0.001). EMAT was negatively associated with serum albumin level (unstandardized *β* = ‐16.42, *p* = 0.011) and positively associated with bPEP/bET (unstandardized *β* = 49.068, *p* = 0.016). LVST was negatively associated with bPEP/bET (unstandardized *β* = ‐125.411, *p* = 0.002), BMI (unstandardized *β* = ‐2.59, *p* = 0.001), and duration of HD (unstandardized *β* = ‐0.003, *p* = 0.032).

## 4. Discussion

This is the first study to assess HD patients before and after HD using acoustic cardiography. Our results showed that S4 and LVST decreased after HD and EMAT increased after HD. In addition, high EMAT/LVST was associated with low albumin, low ABI, and high bPEP/bET, which indicated malnutrition, PAD, and LV dysfunction, respectively.

The advantages of acoustic cardiography include the relatively low cost, noninvasiveness, and ease of use and the ability to recognize LV dysfunction early in HD patients. Moreover, acoustic cardiography is available at the bedside, and cardiographic data of LV function can be obtained faster than with other traditional examination methods. Previous studies have indicated that S3 score can be used in combination with B-type natriuretic peptide to increase the diagnostic accuracy of heart failure [42, 43]. In addition, SDI and S3 score as evaluated using acoustic cardiography have been used to identify heart failure in patients with a reduced ejection fraction with impaired systolic and diastolic function [40]. Nocturnal increases in S4 in asymptomatic older patients are thought to be due to diastolic impairment with increasing age [17]. Acoustic cardiography could therefore be used as a screening bedside tool for the early detection and evaluation of impaired LV systolic function and heart failure in HD patients, especially when echocardiography is not immediately available.

This study investigated changes in acoustic cardiographic markers before and after HD. Shah et al. suggested an association between the strength of S4 and LV stiffness [44]. We found that S4 significantly decreased after HD in all study subjects, including when they were divided into two groups according to median EMAT/LVST. Besides, S3 and the proportion of S3 ≥ 5 had the tendency to decrease after HD. The presence of S3 was associated with LV dysfunction. Fluid overload in dialysis patients has been shown to contribute to LV mass index (LVMI) [45], and Hur et al. indicated that volume control contributed to decreased LVMI and improved blood pressure control [46]. Hemodialysis reduces fluid overload to decrease cardiac load, which may then improve LV stiffness. However, increased EMAT and decreased LVST were found after HD in our study cohort. HD itself may adversely influence the cardiovascular system due to hemodynamic instability and the initiation of systemic inflammation [47, 48]. Jamshidi et al. investigated whether EMAT was responsive to rapid changes in LV systolic function induced by abrupt increases in LV preload. A total of 116 patients were assessed before and after LV angiography with a bolus injection of 40 mL of nonionic contrast dye. In patients with a baseline dP/dt < 1500 mmHg/sec, EMAT decreased from 106 ± 29 ms to 103 ± 18 ms (*p* = 0.02), whereas in patients with a baseline dP/dt > 1500 mmHg/sec, EMAT increased from 88 ± 13 ms to 93 ± 16 ms (*p* = 0.02). These data show an increase in EMAT in the patients whose baseline dP/dt was abnormally low. A directionally opposite change occurred in the patients with a normal baseline dP/dt, probably because of the negative inotropic effect of iopromide, which may also partially explain the opposite finding of EMAT after HD [49]. We proposed that hemodialysis reduces fluid overload to decrease cardiac afterload, which may then improve LV diastolic function. However, it may also reduce cardiac preload and then further decrease LV systolic function. These changes may be different based on different LV functions under the operation of the Starling mechanism. Further studies with more participants are needed to investigate the changes in acoustic cardiographic parameters after HD.

Left ventricular systolic dysfunction leads to heart failure and increased morbidity and mortality in ESRD patients [8, 50]. Previous studies have demonstrated that EMAT, LVST, and EMAT/LVST detected by acoustic cardiography are correlated with echocardiographic evidence to identify LV systolic dysfunction [10, 51–53]. Hypoalbuminemia has also been shown to independently predict heart failure and contribute to worsening heart function in patients with ESRD through malnutrition, myocardial edema, volume overload, oxidative stress, and inflammation [54]. A low serum albumin level is regarded to indicate malnutrition status, and hypoalbuminemia has been associated with reduced LVEF and LV hypertrophy in patients with heart failure [55, 56]. Our results consistently demonstrated that a decreased serum albumin level was associated with increased EMAT/LVST.

HD patients tend to develop structural and functional heart abnormalities due to complex pathophysiological mechanisms [48]. A long-term uremic state has been shown to induce many cardiovascular changes including LV hypertrophy, microvascular disease, myocardial fibrosis, accelerated atherosclerosis, and arteriosclerosis [48]. We found that a low ABI was associated with high EMAT/LVST. Previous studies have demonstrated that the ABI value in patients with LV hypertrophy was significantly lower than that in those without LV hypertrophy and that it was independently and reversely associated with LVMI [57, 58]. Atherosclerosis can directly result in decreased blood perfusion in the lower extremities and increased arterial wall stiffness. This can then contribute to a lower ABI and arterial distensibility and then finally to LV hypertrophy [26, 27, 59]. In contrast, LV hypertrophy can cause LV systolic and diastolic dysfunction and a decrease in cardiac output, which can then further worsen the deficiency of blood perfusion in the extremities and promote the progression of PAD and decease in ABI.

Another important finding of this study is that a high EMAT/LVST ratio was associated with a high bPEP/bET. Increased PEP and shortened ET have been associated with impaired LV systolic function [24, 60], and previous studies have demonstrated that an increased LV mass can result in LV dysfunction and, ultimately, congestive heart failure [61, 62]. Boudoulas et al. reported a significant correlation between increased PEP/ET and increased LVMI in 90 patients with chronic systemic hypertension [28]. We also found that the bPEP/bET was independently positively associated with LVMI and negatively associated with LVEF in our patients with chronic kidney disease. EMAT/LVST can easily be obtained from acoustic cardiography, so it might be helpful for large-volume screening in HD patients to identify those with LV dysfunction.

There are several limitations to this study. First, due to the cross-sectional nature of the study, we could not confirm causal relationships. Further prospective studies are needed to verify our findings. Second, the number of patients in this study was relatively small. Third, the quality of acoustic cardiography data is influenced by exogenous and endogenous noises. Fourth, the patients' acoustic cardiographic parameters were measured only before and after HD, and repeated measurement may be necessary. Fifth, we did not measure ABI after HD, and we will investigate the relationship between the change of ABI and acoustic cardiographic parameters (ACPs) simultaneously before and after the HD in a further study. Finally, the ABI, baPWV, PEP, and ET may not be accurate if the HD patients had upper-limb arterial stenosis. Further prospective studies are warranted to explore the prognostic role of acoustic cardiographic parameters.

## 5. Conclusion

In conclusion, our results demonstrate the changes in acoustic cardiographic parameters before and after HD and that a high EMAT/LVST was associated with low albumin, low ABI, and high bPEP/bET in our HD patients. Acoustic cardiography is a cost-effective and useful tool which may facilitate the timely recognition and treatment of LV systolic dysfunction in asymptomatic HD patients and thereby halt the progression of cardiovascular disease. Further research is warranted to investigate the use of acoustic cardiography in HD patients to predict cardiac outcomes.

## Figures and Tables

**Table 1:** Comparison of baseline characteristics in patients categorized by the median value of EMAT/LVST.

Characteristics	All patients (*n* = 162)	EMAT/LVST > median (*n* = 81)	EMAT/LVST ≤ median (*n* = 81)	*p*
Age	60.4 ± 10.9	59.7 ± 10.5	61.2 ± 11.2	0.412
Male gender (%)	53.1	54.3	51.9	0.753
Smoking (yes)	14.8	18.5	11.1	0.185
Diabetes mellitus (%)	46.3	46.9	45.7	0.875
Hypertension (%)	58.6	58.0	59.3	0.873
Cerebrovascular disease (%)	6.2	6.2	6.2	0.999
Coronary artery disease (%)	9.3	11.1	7.4	0.416
Systolic blood pressure (mmHg)	157.0 ± 23.3	151.2 ± 26.1	156.9 ± 20.2	0.944
Diastolic blood pressure (mmHg)	81.8 ± 13.5	80.9 ± 14.8	82.6 ± 12.1	0.423
Body mass index (kg/m^2^)	24.0 ± 4.0	24.3 ± 4.3	23.7 ± 3.7	0.353
ABI	0.95 ± 0.20	0.93 ± 0.21	0.96 ± 0.18	0.650
baPWV (cm/s)	1957.3 ± 510.9	1938.2 ± 556.7	1975.6 ± 465.5	0.375
bPEP/bET	0.34 ± 0.07	0.35 ± 0.08	0.34 ± 0.06	0.169
Duration of hemodialysis (years)	8.2 (3.9-13.8)	9.2 (4.0-14.9)	7.7 (4.8-11.4)	0.375
Laboratory parameters				
Albumin (g/dL)	3.9 ± 0.3	3.8 ± 0.3	3.9 ± 0.3	0.075
Fasting glucose (mg/dL)	115.4 ± 49.6	118.1 ± 55.2	112.7 ± 43.5	0.490
Triglyceride (mg/dL)	114.0 (85.0-181.0)	113.0 (85.0-170.0)	117.5 (86.0-182.0)	0.626
Total cholesterol (mg/dL)	173.1 ± 41.7	174.7 ± 42.0	171.6 ± 41.6	0.636
High-density lipoprotein (mg/dL)	41.3 ± 11.9	42.1 ± 12.8	40.6 ± 11.0	0.421
Low-density lipoprotein (mg/dL)	89.6 ± 30.0	92.0 ± 31.2	87.2 ± 28.7	0.309
Hemoglobin (g/dL)	10.5 ± 1.3	10.8 ± 1.2	10.3 ± 1.3	0.015
Creatinine (mg/dL)	9.7 ± 2.2	9.6 ± 2.1	9.8 ± 2.2	0.671
CaXP product (mg^2^/dL^2^)	43.8 ± 9.9	45.4 ± 10.3	42.3 ± 9.3	0.054
Uric acid (mg/dL)	7.3 ± 1.4	7.3 ± 1.5	7.3 ± 1.4	0.901
Kt/V (Daugirdas)	1.7 ± 0.3	1.7 ± 0.3	1.7 ± 0.3	0.310
Ultrafiltration (%)	4.7 ± 1.9	4.7 ± 2.3	4.7 ± 1.5	0.985
Acoustic cardiography parameters before hemodialysis				
S3	2.8 ± 1.3	2.9 ± 1.4	2.7 ± 1.1	0.348
S3 ≥ 5 (%)	5.0	5.0	4.9	0.986
S4	4.9 ± 2.0	4.6 ± 2.0	5.1 ± 1.9	0.179
S4 ≥ 5 (%)	26.8	24.7	28.8	0.564
SDI	3.7 ± 1.6	4.3 ± 1.8	3.1 ± 1.1	<0.001
EMAT (ms)	90.9 ± 15.7	101.5 ± 13.5	80.3 ± 9.1	<0.001
EMAT (%)	11.4 ± 2.8	13.2 ± 2.8	9.7 ± 1.4	<0.001
LVST (ms)	331.8 ± 37.5	313.0 ± 37.4	350.6 ± 26.6	<0.001
LVST (%)	42.4 ± 4.6	41.3 ± 5.0	43.6 ± 4.0	0.001

Abbreviation: ABI: ankle-brachial index; baPWV: brachial-ankle pulse wave velocity; bPEP/bET: brachial pre-ejection period/brachial ejection time; CaXP product: calcium-phosphorus product; SDI: systolic dysfunction index; EMAT: electromechanical activation time; LVST: LV systolic time. The median level of EMAT/LVST was 0.27.

**Table 2:** Acoustic cardiography parameters of patients before and after hemodialysis.

Acoustic cardiography parameters	All (*n* = 162)		EMAT/LVST > median (*n* = 81)		EMAT/LVST ≤ median (*n* = 81)	
	Before hemodialysis	After hemodialysis	Before hemodialysis	After hemodialysis	Before hemodialysis	After hemodialysis
S3	2.8 ± 1.3	2.6 ± 1.1	2.9 ± 1.4	2.7 ± 1.2	2.7 ± 1.1	2.5 ± 1.0
S3 ≥ 5 (%)	5.0	1.5	5.0	1.6	4.9	1.4
S4	4.9 ± 2.0	3.9 ± 1.5^∗^	4.6 ± 2.0	3.8 ± 1.6^∗^	5.1 ± 1.9	4.0 ± 1.4^∗^
S4 ≥ 5 (%)	26.8	14.3	24.7	13.8^∗^	28.8	14.7
SDI	3.7 ± 1.6	3.6 ± 1.3	4.3 ± 1.8	4.0 ± 1.4	3.1 ± 1.1	3.3 ± 1.2
EMAT (ms)	90.9 ± 15.7	94.5 ± 15.2^∗^	101.5 ± 13.5	101.6 ± 16.7	80.3 ± 9.1	88.3 ± 10.2^∗^
EMAT (%)	11.4 ± 2.8	11.4 ± 2.8	13.2 ± 2.8	12.7 ± 3.0	9.7 ± 1.4	10.4 ± 2.0^∗^
LVST (ms)	331.8 ± 37.5	319.9 ± 45.5^∗^	313.0 ± 37.4	308.8 ± 36.7	350.6 ± 26.6	329.6 ± 50.1^∗^
LVST (%)	42.4 ± 4.6	39.6 ± 4.5^∗^	41.3 ± 5.0	38.9 ± 4.9^∗^	43.6 ± 4.0	40.2 ± 4.0^∗^

^∗^ *p* < 0.05 compared to patients before hemodialysis. Abbreviations are the same with those in Table 1.

**Table 3:** Determinants of acoustic cardiography parameters of patients before hemodialysis.

Acoustic cardiography parameters	Multivariate (forward)	
	Unstandardized coefficient *β* (95% CI)	*p*
EMAT/LVST		
Albumin	-0.076 (-0.129, -0.024)	0.004
ABI	-0.115 (-0.204, -0.026)	0.011
bPEP/bET	0.278 (0.100, 0.456)	0.003
EMAT		
Albumin	-17.976 (-28.563, -7.390)	0.001
bPEP/bET	53.879 (17.195, 90.563)	0.004
LVST		
ABI	92.032 (50.475, 133.589)	<0.001
bPEP/bET	-122.771 (-207.421, -38.121)	0.005
SDI		
Gender	0.980 (0.433, 1.526)	0.001
Albumin	-1.427 (-2.504, -0.349)	0.010
S4		
Duration of HD	0 (0, 0)	0.002

Adjusting for age, sex, smoking habit, a history of diabetes, hypertension, coronary artery disease and cerebrovascular disease, systolic and diastolic blood pressures, body mass index, ABI, baPWV, bPEP/bET, duration of HD, albumin, fasting glucose, triglyceride, total cholesterol, hemoglobin, creatinine, calcium-phosphorous product, uric acid, Kt/V, and ultrafiltration percent. Abbreviations are the same with those in Table 1.

**Table 4:** Determinants of acoustic cardiography parameters of patients before hemodialysis in patients of ABI ≥ 0.9.

Acoustic cardiography parameters	Multivariate (forward)	
	Unstandardized coefficient *β* (95% CI)	*p*
EMAT/LVST		
bPEP/bET	0.308 (0.161, 0.456)	<0.001
BMI	0.006 (0.003, 0.009)	<0.001
Albumin	-0.042 (-0.091, 0.007)	0.095
Duration of HD	0.000007 (0, 0)	0.011
Creatinine	-0.006 (-0.013, 0)	0.048
EMAT		
Albumin	-16.462 (-29.07, -3.854)	0.011
bPEP/bET	49.068 (9.483, 88.652)	0.016
LVST		
bPEP/bET	-125.411 (-203.446, -47.377)	0.002
BMI	-2.59 (-4.135, -1.044)	0.001
Duration of HD	-0.003 (-0.006, 0)	0.032
SDI		
Systolic blood pressure	0.018 (0.005, 0.03)	0.005
S4		
Duration of HD	0 (0, 0)	0.003

Adjusting for age, sex, smoking habit, a history of diabetes, hypertension, coronary artery disease and cerebrovascular disease, systolic and diastolic blood pressures, body mass index, ABI, baPWV, bPEP/bET, duration of HD, albumin, fasting glucose, triglyceride, total cholesterol, hemoglobin, creatinine, calcium-phosphorous product, uric acid, Kt/V, and ultrafiltration percent. Abbreviations are the same with those in Table 1.

## Data Availability

The data used to support the findings of this study are available from the corresponding author upon request.
